# Effects of Dibutyryl Cyclic-AMP on Survival and Neuronal Differentiation of Neural Stem/Progenitor Cells Transplanted into Spinal Cord Injured Rats

**DOI:** 10.1371/journal.pone.0021744

**Published:** 2011-06-30

**Authors:** Howard Kim, Tasneem Zahir, Charles H. Tator, Molly S. Shoichet

**Affiliations:** 1 Institute of Medical Science, University of Toronto, Toronto, Ontario, Canada; 2 Institute of Biomaterials and Biomedical Engineering, University of Toronto, Toronto, Ontario, Canada; 3 Department of Chemical Engineering and Applied Chemistry, University of Toronto, Toronto, Ontario, Canada; 4 Toronto Western Research Institute and Krembil Neuroscience Centre, University Health Network, Toronto, Ontario, Canada; Biological Research Center of the Hungarian Academy of Sciences, Hungary

## Abstract

Neural stem/progenitor cells (NSPCs) have great potential as a cell replacement therapy for spinal cord injury. However, poor control over transplant cell differentiation and survival remain major obstacles. In this study, we asked whether dibutyryl cyclic-AMP (dbcAMP), which was shown to induce up to 85% *in vitro* differentiation of NSPCs into neurons would enhance survival of transplanted NSPCs through prolonged exposure either *in vitro* or *in vivo* through the controlled release of dbcAMP encapsulated within poly(lactic-co-glycolic acid) (PLGA) microspheres and embedded within chitosan guidance channels. NSPCs, seeded in fibrin scaffolds within the channels, differentiated *in vitro* to betaIII-tubulin positive neurons by immunostaining and mRNA expression, in response to dbcAMP released from PLGA microspheres. After transplantation in spinal cord injured rats, the survival and differentiation of NSPCs was evaluated. Untreated NSPCs, NSPCs transplanted with dbcAMP-releasing microspheres, and NSPCs pre-differentiated with dbcAMP for 4 days *in vitro* were transplanted after rat spinal cord transection and assessed 2 and 6 weeks later. Interestingly, NSPC survival was highest in the dbcAMP pre-treated group, having approximately 80% survival at both time points, which is remarkable given that stem cell transplantation often results in less than 1% survival at similar times. Importantly, dbcAMP pre-treatment also resulted in the greatest number of *in vivo* NSPCs differentiated into neurons (37±4%), followed by dbcAMP-microsphere treated NSPCs (27±14%) and untreated NSPCs (15±7%). The reverse trend was observed for NSPC-derived oligodendrocytes and astrocytes, with these populations being highest in untreated NSPCs. This combination strategy of stem cell-loaded chitosan channels implanted in a fully transected spinal cord resulted in extensive axonal regeneration into the injury site, with improved functional recovery after 6 weeks in animals implanted with pre-differentiated stem cells in chitosan channels.

## Introduction

Repair after traumatic spinal cord injury (SCI) remains a significant challenge. Extensive cell death and tissue disorganization, including demyelination, coupled with an inherent aborted and lack of spontaneous regeneration results in permanent disruption of signalling pathways. Although resident endogenous neural stem cells exist lining the central canal of the adult spinal cord [1], their recruitment is limited in response to injury [2]. This is in large part due to the inhibitory environment of adult central nervous system (CNS) tissue, which is further exacerbated after injury.

Cell transplantation therapy is a promising approach to replace the damaged or lost cells after SCI. In particular, adult neural stem/progenitor cells (NSPCs) are attractive because of their ability to form the three major CNS cell types exclusively. However, in practice, NSPCs transplanted into the spinal cord have a much greater tendency to differentiate into either astrocytes or oligodendrocytes, and rarely undergo neuronal differentiation in vivo [3], [4], [5]. Indeed, many attempts to program NSPCs to preferentially promote neuronal differentiation *in vitro* have not succeeded *in vivo* in the spinal cord [6], [7], [8]. It is important to investigate the potential of neuronal replacement strategies for SCI as neurons are the core functional component of spinal cord signalling. Neuronal replacement after SCI is important to areas rich in neurons such as the cervical or thoraco-lumbar segments of the spinal cord.

The advantages of transplanting NSPCs in combination with implants of biomaterial guidance channels have been described [9], [10], [11]. After complete spinal cord transection injury in the rat, new tissue formed between the two spinal cord stumps as early as five weeks post injury [10]. Transplanted NSPCs were found to survive and integrate into this tissue bridge, and were distributed throughout the length of the bridge. However, very few NSPCs differentiated into neurons and limited functional recovery occurred.

Directed differentiation of NSPCs can be accomplished through exposure to specific soluble factors. For example, platelet-derived growth factor has been shown to promote oligodendrocyte fate-specification [12] while bone morphogenetic protein-2 promotes astrocyte differentiation [13]. Zahir et al., screened several factors for promoting neurogenesis from adult rat subventricular zone (SVZ)-derived NSPCs and reported that dibutyryl cyclic-AMP (dbcAMP), a membrane-permeable analogue of cyclic-AMP, and the cytokine interferon-gamma both enhanced neuronal differentiation after one week exposure in culture [14]. Several other groups have used dbcAMP to differentiate a variety of neural stem or progenitor cells into neurons [15], [16], [17], [18]. This is thought to be primarily through the PKA pathway [15], which causes upregulation of cAMP-responsive element binding (CREB), an important transcription factor in regulating neuronal activity [19]. Moreover, dbcAMP may have beneficial effects independent of its action on NSPCs, particularly with respect to promoting axonal regeneration [20]. It is a downstream molecule in neurotrophin signaling [21], [22], which has been shown to allow neurite outgrowth in the presence of myelin inhibitors *in vitro* [21] and *in vivo* [23].

In the present study, directed neuronal differentiation of NSPCs with dbcAMP was characterized first *in vitro* on 2D chitosan film surfaces and 3D fibrin scaffolds, and then *in vivo* in a rat spinal cord transection model. Specifically, NSPCs were transplanted in chitosan guidance channels in which the walls were loaded with dbcAMP-eluting poly(lactic-co-glycolic acid) (PLGA) microspheres [24]. We have recently shown that chitosan channels exhibit excellent biocompatibility in the spinal cord over a one year period [25]. Microsphere-based release of dbcAMP facilitates local and sustained drug release, providing a potential method for *in situ* differentiation. The effect of directed neuronal differentiation of NSPCs with dbcAMP was evaluated for its effect on survival, differentiation, and integration into the rat spinal cord following SCI. As well, the timing of differentiation was also investigated: NSPCs were either pre-differentiated *in vitro* prior to transplantation, or transplanted with dbcAMP-releasing microspheres for *in vivo* differentiation. Finally, fibrin was used as a 3D scaffold to entrap/suspend the cells within the channels. Fibrin hydrogel has previously shown to be useful for stem cell transplantation to the spinal cord [26].

## Materials and Methods

### Chitosan Films and Channels

High quality chitosan (Protosan UP B 80/20; NovaMatrix) was dissolved as a 3 wt% solution in 2% acetic acid, and then diluted with an equal volume of ethanol. Chitosan films were prepared by allowing 10 ml of chitosan solution to air dry in a 10 cm Petri dish. Once dry, the film was soaked in neutralization solution (90% methanol, 7% water, 3% ammonium hydroxide) for 15 min then washed thoroughly with water. Films were cut into 8 mm discs and disinfected in 70% ethanol prior to use in cell culture studies.

Chitosan channels were prepared as previously described [27]. Briefly, chitosan/ethanol solution was reacted with acetic anhydride and injected between concentric cylindrical glass molds, resulting in chitin hydrogel channels with an inner diameter of 4 mm and outer diameter of 8 mm. Chitin channels were washed for 24 h then were hydrolyzed in 40 wt% NaOH at 110°C for 2 h, rinsed, then hydrolyzed again for another 25 min resulting in chitosan with a degree of deacetylation of 85% as assessed by ^1^H-NMR [28]. Upon washing, channels were air-dried on 3.7 mm stainless steel rods. Channels were cut into 8 mm lengths.

PLGA microspheres were embedded along the inner walls of chitosan channels as previously described [24]. Briefly, 5 mg of blank or dbcAMP-containing PLGA microspheres (described later), was suspended in 25 µl of a buffered chitosan solution (1∶5, v/v mixture of 2% chitosan in 1% acetic acid and 75 mM phosphate buffer, pH 7). The microsphere solution was introduced to the interior of channels under a rotation of 2500 rpm and spun until dry. The microsphere-embedded channels were then neutralized in a solution of 90% methanol, 7% water, and 3% ammonium hydroxide for 1 min, rinsed thoroughly, and air dried. Channels were then sterilized by gamma irradiation at a dose of 2.5 MRad prior to use in subsequent in vitro or in vivo cell culture studies.

### Dibutyryl Cyclic-AMP PLGA Microspheres

Poly(lactic-co-glycolic acid) (PLGA) 50/50 of both 0.20 and 0.37 g/dl inherent viscosity in hexafluoroisopropanol (HFIP) were used as received from Lactel. PLGA microspheres were fabricated using a W/O/W double emulsion procedure. The inner aqueous phase consisted of 75 µl of ddH20 with or without 40 mg dibutyryl cyclic-adenosine monophosphate (Sigma-Aldrich). 130 mg of PLGA (80 wt% 0.20 g/dl, 20 wt% 0.37 g/dl) was dissolved in 600 µl of dichloromethane/acetone (3∶1). The two solutions were emulsified under sonication (Vibracell VCX 130, Sonics and Materials) for 45 s using a 3 mm probe at 30% amplitude. This emulsion was added to 25 ml of 2.5% polyvinyl alcohol (PVA)/10% NaCl solution and homogenized at 4000 rpm for 60 s. The resultant solution was poured into a 250 ml bath of 0.25% PVA/10% NaCl solution under magnetic stirring. In a typical batch, three identical preparations were added to the final bath. Four hours later, the hardened microspheres were collected and washed by centrifugation, then washed again over a 0.2 µm filter, collected, lyophilized and stored at −20°C until use.

The amount of encapsulated dbcAMP was measured by dissolving a known quantity of microspheres in 800 µl of dichloromethane. An equal volume of water was added and the solution was vortexed and centrifuged for 3 min at 10 000 rpm. The water phase containing drug was collected. This process was repeated two more times. The collected water phase was then measured for dbcAMP content using a Nanodrop UV spectrometer at 273 nm against a calibration curve of known dbcAMP solution concentrations. Encapsulation efficiency (EE) was calculated as the percentage of actual loading compared to the maximum theoretical loading based on preparation parameters.

To measure the release of dbcAMP from microspheres or microsphere-loaded channels, approximately 3 mg of microspheres (accurately measured) or a single microsphere-loaded channel was suspended in 1.8 ml PBS and stored under gentle agitation at 37°C. At various times, 2 µl of the release buffer was sampled for measurement by Nanodrop UV spectroscopy at 273 nm. Known concentrations of dbcAMP were incubated alongside release samples, and a small but measurable decrease in dbcAMP signal due to hydrolysis was observed over time and was accounted for in drug release calculations. At the end of the release period, mass balance measurement of the remaining dbcAMP in microspheres was quantified by phase extraction in dichloromethane.

### Adult Neural Stem/Progenitor Cells

Primary NSPCs were isolated from the subventricular zone of adult Wistar rats, both GFP and non-GFP, and propagated as previously described [14]. The transgenic rats expressing GFP (Wistar-TgN(CAG-GFP)184ys) were the gift of Dr. Armand Keating (University of Toronto, Toronto) and have been previously described [29], [30]. NSPCs were passaged as neurospheres in growth media: Neurobasal media (Gibco-Invitrogen), B27 neural supplement (Gibco-Invitrogen), 2 mM L-glutamine (Sigma-Aldrich), 100 µg/ml penicillin-streptomycin (Sigma-Aldrich), 20 ng/ml epidermal growth factor, 20 ng/ml basic fibroblast growth factor (Sigma-Aldrich), and 2 µg/ml heparin (Sigma-Aldrich). NSPCs were used after the fourth or fifth passage for *in vitro* and *in vivo* studies.

### Chitosan Film Studies

Chitosan films were placed in 48-well plates and soaked in media prior to use. NSPCs were spun down at 1500 rpm for 5 min and the resultant pellet was re-suspended and dissociated in fresh growth media, with or without dbcAMP. The cell solution was then added so that each well contained 30 000 cells, and the final volume of media in the wells was 500 µl. After 24 h, the media was removed and replaced with differentiation media - Neurobasal media containing B27 supplement, L-glutamine, penicillin-streptomycin, and 1% FBS - with or without dbcAMP. Half-volume media changes were performed every 48 h thereafter.

### Chitosan Channel Studies

For cell culture studies in channels, NSPCs were seeded in three-dimensional fibrin scaffolds. Fibrinogen (F3879; Sigma) was dissolved as a 22.2 mg/ml solution in 0.03 M Tris buffered saline (TBS), pH 7, filtered through a 0.22 µm filter, and then stored at −80°C. Thrombin (T6884; Sigma) and aprotinin (A4529; Sigma) were similarly dissolved in TBS at concentrations of 24 NIHU/ml and 5 mg/ml respectively, filtered and stored at −80°C.

On the day of use, fibrinogen, aprotinin, and laminin (Sigma) solutions were combined, resulting in a solution of 20 mg/ml fibrinogen, 500 µg/ml aprotinin, and 20 µg/ml laminin. This solution was mixed 5∶3 with media containing dissociated NSPCs. The cell-fibrinogen mixture (48 µl) was then added by pipette into the lumen of pre-hydrated chitosan channels, followed by 12 µl of thrombin solution. After mixing, the fibrin scaffold containing NSPCs formed within thirty seconds. The initial cell number in each fibrin-filled channel was 250 000.

Cell-seeded channels were then placed in 24-well plates containing 1 ml of fresh growth media, with or without 1 mM dbcAMP. For *in* vitro studies, the media was removed after 24 h and replaced with differentiation media, with or without 1 mM dbcAMP. Half-volume media changes were performed every 48 h thereafter.

NSPC-seeded channels for in vivo studies were prepared similarly using GFP-positive cells. Channels were implanted after either 1 or 4 days in vitro (1div and 4div respectively), depending on dbcAMP treatment (Table 1). The 1div channels did not receive a media exchange prior to transplantation. The 4div channels received media changes as described above, but FBS was removed from the differentiation media. Excess samples from each treatment group were prepared and fixed on the day of transplant and analyzed for cell number and percentage of betaIII-positive neurons.

**Table 1:** *In vivo* treatment groups.

Group	Culture period prior to transplant (days)	1 mM dbcAMP in media during culture period?	Microsphere Content	Cell Number on Day of Transplant (×10^3^)	Animals for two-week survival (n)	Animals for six-week survival (n)
Untreated, 1div	1	No	No drug	83±4	5	5
Untreated, 4div	4	No	No drug	46±8	3	-
dbcAMP-MS, 1div	1	No	dbcAMP	78±12	4	5
dbcAMP, 4div	4	Yes	No drug	57±10	4	6

### Immunocytochemistry

For cell culture experiments, fixed samples were permeabilized with PBS containing 0.3% Triton X-100 and 1% bovine serum albumin (BSA) for 15 min followed by 1 h blocking in 1% BSA. Samples were incubated for 2 h with primary antibodies including mouse anti-betaIII tubulin (1∶1000, Covance) for neurons, mouse anti-GFAP (1∶200, Millipore) for astrocytes, mouse anti-RIP (1∶5, DSHB) for oligodendrocytes, mouse anti-nestin (1∶100, BD Biosciences) for neural precursors, and mouse anti-Ki67 (1∶100, Novocastra Laboratories Ltd) for proliferating cells. Following three 10 min washes in PBS, samples were incubated with goat anti-mouse IgG Cy3 (1∶500, Jackson Immunoresearch) for 1 h. After another series of washes, samples were incubated with 10 µM Hoecsht 33342 (Invitrogen) nuclear dye and coverslipped with Vectashield mounting media. A minimum of five randomly selected fields per sample were photographed on an Olympus BX61 epifluorescent microscope to quantify cells co-labelled with nuclear dye and secondary antibody.

### Quantitative RT-PCR

Cell culture samples were lysed after seven days and RNA was collected and purified using RNAqueous Micro kit (Ambion). Following RNA isolation and DNAse I treatment, total RNA concentration and purity was measured using Nanodrop ND-1000. RNA concentration was normalized across samples then built into cDNA libraries using a AffinityScript RT kit (Stratagene).

Quantitative reverse transcriptase-polymerase chain reaction (qRT-PCR) amplification was performed on a Light Cycler 480 (Roche) using SYBR Green II master mix kit (Roche). Following a 5 min activation at 95°C, fluorescent measurements were taken after temperature cycles of 10 s at 90°C, 15 s at 65°C, and 10 sec at 72°C. After 50 cycles, the temperature was held at 65°C for 1 min, followed by temperature ramping to 95°C over 8 min to generate a melt curve. All qRT-PCR samples were performed in triplicate. Gene expression for each sample was normalized to the housekeeping gene HPRT. Primer design for target genes of interest are previously reported [31]. Data is expressed as fold-difference compared to undifferentiated (day 0) cells.

### Animal Studies

The animal work was approved by the Animal Care Committee of the University Health Network (Protocol No. AUP 125.15). Adult female Sprague-Dawley rats (250–350 g, Charles River, St. Constant, QC) were anesthesized with 4% isofluorane and an oxygen/nitrous oxide (2∶1) mixture. The isofluorane concentration was dropped to 2% during the operation. Following incision of the skin on the back, vertebral levels T6 to T10 were exposed. Laminectomy was performed on T7 to T9 exposing the spinal cord. The facets of the vertebrae at T7 to T9 were also removed. A midline longitudinal durotomy was performed, then cut laterally at the rostral and caudal ends to fully expose the underlying spinal cord. The spinal cord was completely transected at T8 with microscissors, as were the adjacent underlying dorsal and ventral roots.

Chitosan channels containing NSPCs in the fibrin scaffold were then implanted by placing the transected stumps into the channel openings. Treatment groups are described in Table 1. Channels were positioned symmetrically such that the caudal and rostral stumps were inserted equidistant inside the channel, approximately 2 mm, so that the gap between the stumps was approximately 4 mm. The spinal cord stumps were inserted sufficiently far into the channel so that the stumps were in apposition to the fibrin scaffolds within the channels. A 4 mm×3 mm sheet of Gore-Tex Preclude MVP membrane (Gore-Tex, Flagstaff, AZ) was placed at each end covering the channel-spinal cord interface. 30 µl of fibrin glue (Beriplast P, provided generously from CSL Behring) was then applied to secure the stumps to the implant. The overlying muscle and skin were closed with 3-0 Vicryl sutures (Ethicon) and metal clips, respectively.

Rats were given buprenorphrine post-surgery, and every 8–12 h for the next 48 h. Immunosuppression with cyclosporine-A (15 mg/kg) was administered daily via subcutaneous injection. Functional recovery was assessed using the Basso, Beattie, and Bresnahan (BBB) open field locomotor scale [32] once a week. The scores for the left and right hindlimb were averaged for each animal at each timepoint.

### Tissue Preparation

At either 2 or 6 weeks post-implantation, animals were sacrificed via transcardial perfusion with 4% paraformaldehyde in 0.1 M PBS, pH 7.4. Spinal cords were removed and cryoprotected in 30% sucrose for a minimum of 24 h. The length of cord encompassing the implant site, as well as 2–3 mm on either side, was removed for sectioning. Tissue was cut as 20 µm cryosections onto Superfrost Plus slides (Fisher Scientific, Markham, ON) in 1∶10 series and stored at −80°C. For the 6 week animals, one sample from each treatment group was cut parasaggitally. All other 6 week samples, and all 2 week samples, were cut as cross-sections.

### Tissue analysis

To quantify cell survival, every tenth section was stained with DAPI (Vectashield) and imaged under fluorescence to detect the GFP-positive transplanted cells. Cell counts were calculated by manually counting GFP-positive cells associated with DAPI-stained nuclei across the sample and extrapolating by a factor of ten. False positive signals associated with autoflourescence were eliminated by also imaging under the Cy3 filter.

For immunohistochemical analysis of tissue sections, slides washed with PBS then blocked in 2% goat serum and 0.3% Triton X-100 for 1 h. Primary antibodies were incubated in block or PBS overnight at 4°C. After three consecutive 10 min PBS washes, slides were incubated with secondary antibody in PBS for 1 h. Following another 3 washes, sections were coverslipped with Vectashield anti-fade mount containing DAPI. Some sections were double-labelled. In these cases the second primary-secondary antibody pairings was stained following the first pairing.

Primary antibodies used were mouse and rabbit anti-betaIII tubulin (1∶1000, Covance), mouse anti-βIII tubulin (1∶1000, Covance) for neurons, mouse anti-GFAP (1∶200, Millipore) for astrocytes, mouse anti-CC1 (1∶1000, Calbiochem) for oligodendrocytes, mouse anti-nestin (1∶100, BD Biosciences) for neural precursors, mouse anti-3CB2 (1∶50, Hybridoma Bank) for radial glia, mouse anti-Reca1 (1∶100, Serotec) for endothelial cells, mouse anti-prolyl-4-hydroxylase (1∶100, Millipore) for fibroblasts, mouse anti-GFP (1∶400, Invitrogen) for green fluorescent protein, and mouse anti-Ki67 (1∶100, Novocastra Laboratories Ltd) for proliferating cells. Secondary antibodies included goat anti-mouse Alexa 568 (Invitrogen), goat anti-rabbit Alexa 568 (Invitrogen), goat anti-mouse Alexa 647 (Invitrogen), and goat anti-mouse Cy5 (Jackson Immuoresearch) all at 1∶500. Images for differentiation analysis were taken on a Zeiss Observer Z1 confocal microscope using Volocity 4.3.2 software. A minimum of ten random fields containing the GFP-positive transplanted NSPCs were photographed over multiple tissue sections for each animal per stain. Phenotypic analysis of transplanted NSPCs was calculated based on identification of GFP-positive cells associated with a DAPI-stained nucleus and the immunohistological marker of interest.

### Statistics

Comparisons involving one independent factor (eg. treatment) or two independent factors (eg. treatment and time) were analyzed using one-way and two-way ANOVA, respectively, followed by Bonferroni post-hoc testing. P-values less than 0.05 were used as the criteria for statistical significance. Statistical analysis was performed using GraphPad Prism software. All values are represented as mean ± standard deviation.

## Results

### Dibutyryl Cyclic-AMP Promotes Neuronal Differentiation of NSPCs

**Figure 1:**
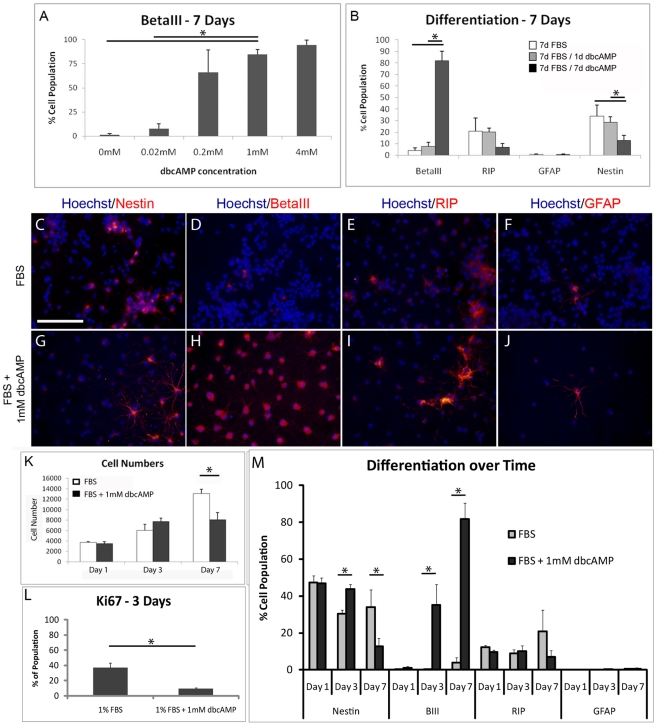
NSPCs respond to dbcAMP *in vitro*. **A**) Dose response curve of dbcAMP on neuronal differentiation of NSPCs after 7 days in culture. **B**) Differentiation profile of NSPCs after 7 days. NSPCs were treated with media containing 1 mM dbcAMP for 0, 1 or 7 days. Only sustained exposure to dbcAMP resulted in increased number of BetaIII-positive neurons. **C–J**) Representative images of NSPCs after 7 days in culture for markers of progenitors cells (nestin), neurons (BetaIII), oligodendrocytes (RIP), and astrocytes (GFAP). Scale bar represents 100 µm. **K**) Cell numbers at 1, 3, and 7 days in culture with or without 1 mM dbcAMP. **L**) Ki67 staining for proliferating cells after 3 days. **M**) Cell differentiation over time with or without 1 mM dbcAMP treatment. Data represented as mean ± standard (n = 3 to 9). Statistical differences denoted by *, p<0.05.

NSPCs were cultured on chitosan films and exposed to varying concentrations of dbcAMP for 7 d, then stained with the neuronal marker betaIII tubulin to quantify its effect on directed neuronal differentiation (Figure 1A). In the absence of dbcAMP, NSPCs produced very few neurons (1.4±1.2%). At increasingly higher dbcAMP concentrations, the percentage of betaIII tubulin-positive cells also increased, reaching as high as 94.5±0.5% with 4 mM dbcAMP. 1 mM dbcAMP, which produced 84.8±4.8% neurons, was selected as the target concentration for subsequent studies because it resulted in greater numbers of total cells compared to 4 mM.

A more complete differentiation profile was performed on NSPCs treated with dbcAMP (Figure 1B–J). NSPCs were cultured for 7 d with either no dbcAMP, 1 mM dbcAMP for the first day only, or 1 mM dbcAMP for all 7 days. All groups contained 1% FBS in the media. Only with prolonged exposure to dbcAMP was extensive neuronal differentiation seen. This was in contrast to 1 d dbcAMP exposure which resulted in only 7.7±3.6% neurons. The increase in neurons with extended dbcAMP treatment was accompanied by a decrease in the percentages of RIP-positive oligodendrocytes (7.0±3.4% vs. 21.1±11.4% in FBS alone) and nestin-positive progenitor cells (12.7±4.5% vs. 33.9±9.6% in FBS alone). Very few GFAP-positive astrocytes were produced, with or without dbcAMP, as is typical for our population of brain-derived rat NSPCs [12], [14], particularly on when cultured chitosan substrate [31]. One day treatment with dbcAMP had no effect on differentiation compared to FBS controls.

Cell density was notably lower in dbcAMP-treated wells after 7 d, suggesting either cytotoxic or anti-proliferative effects of the drug. To investigate this, NSPCs were characterized at 1, 3, and 7 d for total cell number, as well as for markers of proliferation and differentiation (Figure 1K,L). Prolonged exposure to dbcAMP in culture had no effect on total cell numbers after 3 d, but there was a clear anti-proliferative effect by 7 d where dbcAMP largely promoted post-mitotic neuron formation. Ki67 staining for proliferating cells was 4-fold lower for dbcAMP-treated cells (9.4±1.4%) at 3 d than controls (37±6.4%). DbcAMP appeared to have a direct effect on promoting neurons vs. being selectively cytotoxic to glial cells because oligodendrocyte and astrocyte populations did not decrease over time (Figure 1M).

### PLGA Microspheres for dbcAMP Release

**Figure 2:**
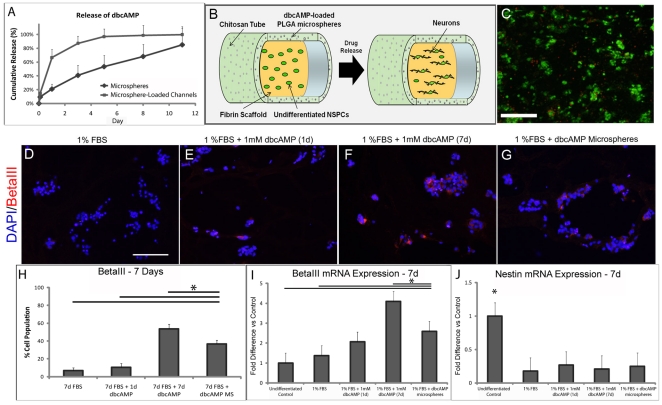
Microsphere-loaded channels effectively release dbcAMP *in vitro*. **A**) Cumulative release profiles of dbcAMP from free-floating microspheres and microsphere-loaded channels. The process of embedding microspheres into channel walls is likely responsible for early degradation of PLGA and faster drug release from channels. **B**) Schematic of the entubulation strategy. NSPCs are seeded on fibrin scaffold within a chitosan channel. Drug-loaded PLGA microspheres release the differentiation factor dibutyryl cyclic-AMP in a local and sustained manner, influencing NSPCs to preferentially differentiate into neurons. **C**) Viability of NSPCs in a three-dimensional fibrin scaffold. Simultaneous staining of CalceinAM (green) and Ethidium homodimer (red) for live and dead cells respectively show good cell viability of NSPCs in fibrin scaffolds at 1 week. Scale bar represents 100 µm. **D–G**) Immunostaining of NSPCs for DAPI-nuclear stain and betaIII-tubulin with various dbcAMP treatments. Scale bar represents 100 µm. **H**) Quantification of betaIII-tubulin immunostained NSPCs with various dbcAMP treatments. **I,J**) Quantitative RT-PCR data for (I) betaIII tubulin and (J) nestin mRNA expression with various dbcAMP treatments, normalized to housekeeping gene HPRT. Data represented as mean ± standard (n = 3 to 6). Statistical differences denoted by *, p<0.05.

Based on the *in vitro* data where the greatest neuronal differentiation was observed in the presence of constant dbcAMP for 7 d, dbcAMP was encapsulated in PLGA microspheres and the release from which was tailored for 1 week. The encapsulation efficiency of dbcAMP was 80% and the loading was 19 wt%. Release curves of dbcAMP from PLGA microspheres, both free-floating in buffer and embedded within chitosan channels, are shown in Figure 2A. Drug release from native microspheres was linear over 11 days. Once incorporated into channels, the release of dbcAMP occurred over approximately 5 days, after which the drug contents are depleted. This discrepancy in release profiles is largely due to drug losses during the process of embedding microspheres into the channel. This was confirmed by mass balance which showed less drug content in dbcAMP microsphere-loaded channels than expected based on microsphere quantity.

### NSPC Differentiation in Microsphere-Loaded Channels

DbcAMP-loaded microspheres were incorporated into chitosan channels and tested for their efficacy in differentiating NSPCs in three-dimensional constructs, as depicted in Figure 2B. NSPCs were seeded into fibrin scaffolds and cultured within microsphere-loaded channels containing either no drug or dbcAMP. After seven days, NSPCs in three-dimensional fibrin scaffolds were well distributed and viable as assessed by calceinAM (live)/ethidium homodimer (dead) staining (Figure 2C).

NPSCs remained responsive to dbcAMP in 3D fibrin scaffolds (Figure 2D–H). NSPC cultured in media containing 1 mM dbcAMP for 7 days resulted in 54±5% betaIII-positive neurons, while NSPCs cultured with dbcAMP-releasing microspheres resulted in approximately 37±4% neurons. Both groups had significantly greater percentages of neurons than NSPCs cultured without dbcAMP, or dbcAMP for only the first 24 h, which resulted in only 7±3% and 11±4% neuronal differentiation, respectively. Quantitative RT-PCR measurement of betaIII-tubulin mRNA showed a similar trend of expression between treatment groups, further supporting the immunostaining data (Figure 2I,J).

### *In vivo* NSPC Transplantation

NSPCs were seeded in the fibrin scaffold within channels and incubated 1 or 4 days in vitro (div) prior to transplantation (see Table 1). The 1 div samples included NSPCs seeded in channels containing blank or dbcAMP-releasing microspheres. These groups were transplanted after only one day incubation to ensure that the majority of dbcAMP released from microspheres occurs *in vivo*. The 4 div groups included both NSPCs pre-differentiated (40% betaIII-tubulin-positive neurons) in 1 mM dbcAMP media prior to transplant and the corresponding untreated control. Cell numbers at the time of transplantation are listed in Table 1. The decrease in cell numbers for the 4 div groups was attributed to incubation in serum-free media.

**Figure 3:**
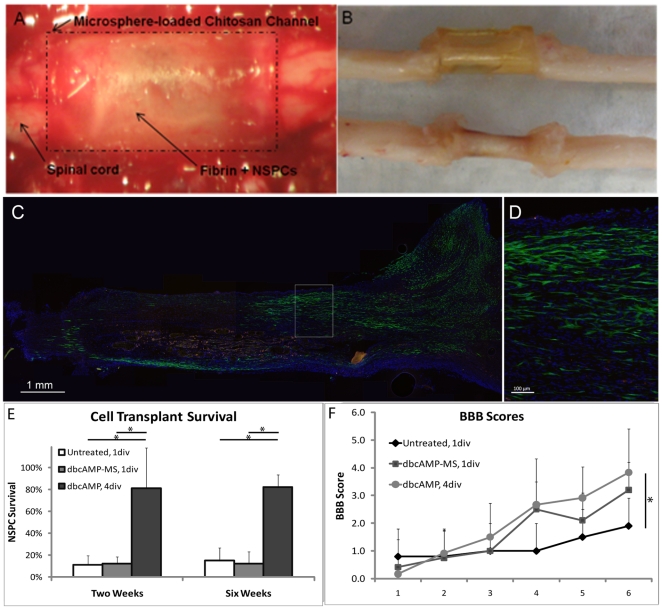
Channel implantation after spinal cord transection facilitates tissue bridging, NSPC survival, and behavioural improvement over time. **A**) Photograph of the surgical implantation of fibrin-filled chitosan channels. **B**) Tissue bridges obtained from animals 2 weeks after implantation. **C,D**) Longitudinal section of tissue bridge demonstrating NSPC survival after 6 weeks in an animal receiving dbcAMP pre-treatment (dbcAMP, 4div). Boxed area in (C) is magnified in (D). **E**) NSPC survival after 2 and 6 weeks for various treatment groups. **F**) Assessment of functional recovery using the BBB locomotor scale. After 6 weeks, rats receiving transplants of dbcAMP-pre-treated NSPCs show a statistically significant increase in hindlimb function relative to untreated animals (*, p<0.05). Mean ± standard deviation shown for n = 4 to 6.

The channels were implanted following spinal cord transection, with the spinal cord ends abutting the ends of the fibrin scaffold (Figure 3A). After two and six weeks, animals were sacrificed. Robust tissue bridges across the injury site formed as early as two weeks (Figure 3B). As shown in Figures 3C and D, GFP-positive transplanted NSPCs were found throughout the bridge tissue. Cell transplant survival was assessed by GFP-positive cell counts (Figure 3E). At 2 weeks, NSPCs pre-treated with free dbcAMP resulted in significantly higher cell survival, approximately 80%, compared to both untreated NSPCs (1 div or 4 div) and NSPCs implanted in chitosan channels with dbcAMP microspheres. Cell survival percentages did not differ between two and six weeks for any treatment group. Staining for the proliferative marker Ki67 was very low for NSPCs at two weeks (data not shown). There was no definite migration of NSPCs into the spinal cord stumps.

Six week animals were also monitored weekly for hindlimb function using the BBB motor function scale. A small but statistically significant increase in hindlimb movement at 6 weeks was observed in animals receiving NSPCs pre-treated with dbcAMP for 4 div compared to untreated NSPCs (Figure 3F).

**Figure 4:**
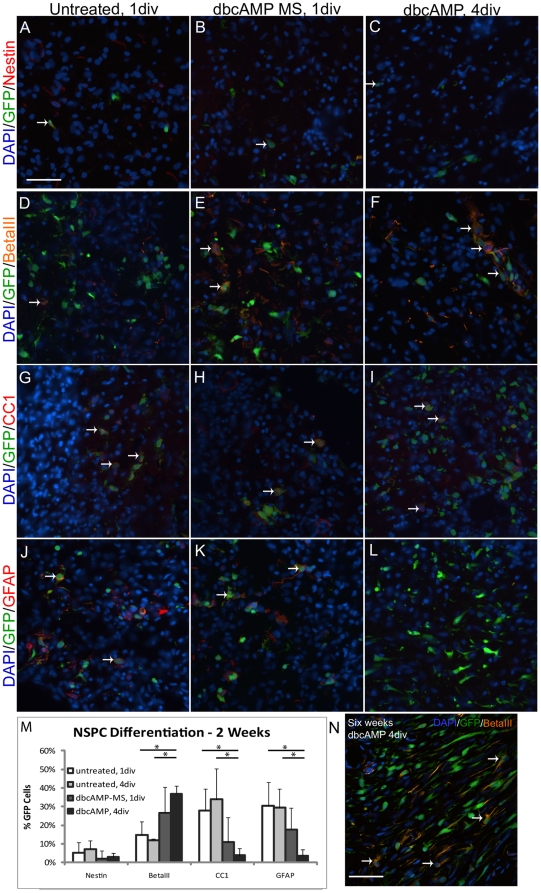
Differentiation profiles of NSPCs are impacted by dbcAMP treatment. **A–L**) Representative images of tissue samples demonstrating NSPC differentiation profile of (A–C) nestin-positive progenitor cells, (D–F) BetaIII-positive neurons, (G–H) CC1-positive oligodendrocytes, and (J–L) GFAP-positive astrocytes. Scale bar represents 50 µm. **M**) Quantification of NSPC differentiation profile for the various treatment groups. Mean ± standard deviation are plotted, n = 3 to 5; significant differences noted with an asterisks, p<0.05. **N**) Deconvoluted confocal image of betaIII-positive NSPC-derived neurons (arrows) 6 weeks post-transplantation. Scale bar represents 50 µm.

Differentiation of NSPCs after two weeks was examined and the results are summarized in Figure 4. All four groups expressed low levels of nestin showing that few NSPCs remained as uncommitted progenitors. BetaIII-tubulin staining show about 15% neuronal differentiation for NSPCs not exposed to dbcAMP (both 1 div and 4 div). NSPCs pre-treated with dbcAMP resulted in 37.0±4.1% neurons after two weeks, similar to time-of-transplant values. Treatment with dbcAMP microspheres resulted in an intermediate percentage of neurons, 26.7±13.9%. CC1-staining for oligodendrocytes was highest for untreated NSPCs (27.9±11.7% and 33.8±16.5% for 1 div and 4 div, respectively) and lowest for dbcAMP pre-treated NSPCs (3.9±3.8%). Similarly, GFAP-staining for astrocytes was also highest for untreated NSPCs (30.3±12.8% and 29.3±10.3% for 1 div and 4 div, respectively) and lowest for dbcAMP pre-treated NSPCs (3.5±3.6%). DbcAMP-microsphere treated NSPCs resulted in intermediate percentages of oligodendrocytes and astrocytes (10.9±13.2% and 17.7±11.6%, respectively) which were not significantly different from either untreated or pre-treated groups. Staining for mature neuronal markers MAP2 and NeuN were negative across all groups. Transplanted NSPCs were also negative for the radial glia marker 3CB2.

At six weeks, despite similar cell survival numbers, the majority of transplanted NSPCs had lost expression of the typical CNS phenotypic markers used above, independent of treatment. Staining with an antibody against the GFP-antigen resulted in positive staining (data not shown), indicating that tissue preparation was not an issue. Large populations of unclassified NSPCs have been reported previously [5], [9]. It is interesting to note that, particularly in the dbcAMP-pre-treated animals, the surviving NSPCs seemed to form continuous networks across the bridge, with highly ordered longitudinal orientation (Figure 3C,D). Cell morphology suggests a more mature phenotype with elongated cell bodies and processes. A small subset of the dbcAMP pre-treated cells were betaIII-tubulin positive at six weeks (Figure 4N), although staining with mature neuronal markers MAP2 and NeuN were largely negative.

**Figure 5:**
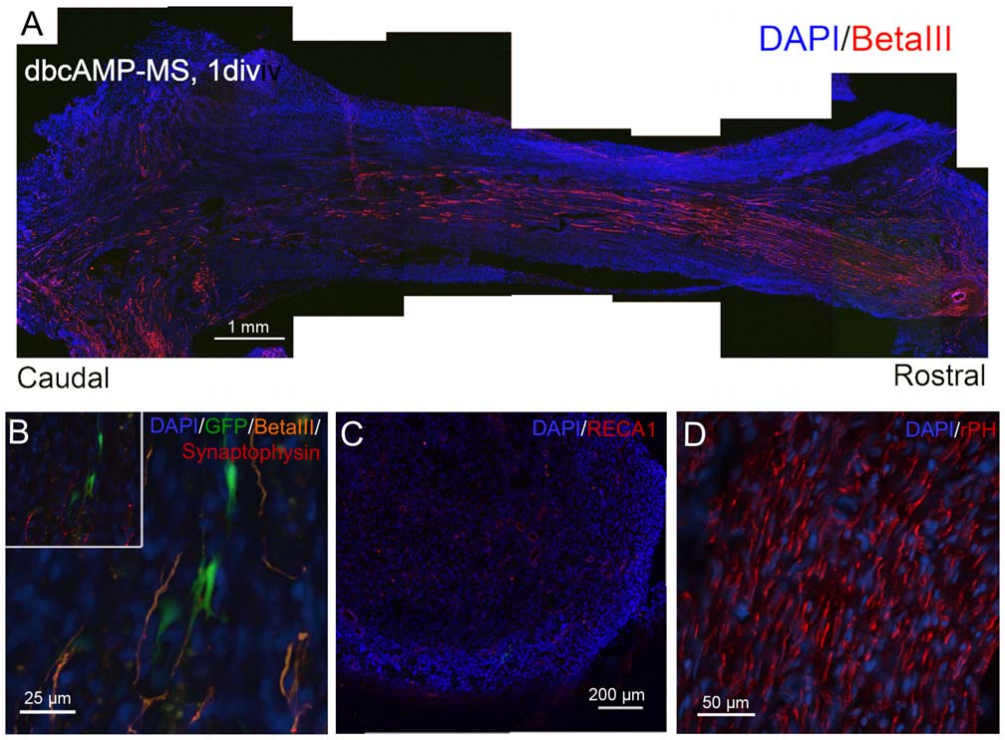
The regenerated bridge tissue contains host axons, blood vessels, and fibroblasts. **A**) Representative image of endogenous axonal regeneration into the tissue bridge based on betaIII tubulin staining. **B**) Evidence of association between betaIII-positive endogenous axons with surviving GFP-positive NSPCs at six weeks. Synaptophysin staining is observed at the interface (inset). **C**) RECA1 staining for endothelial cells show blood vessel formation throughout the tissue bridge at 2 weeks. **D**) Prolyl-4-hydroxylase (rPH) staining of bridge tissue indicates that the majority of cells are collagen producing fibroblasts.

Axonal regeneration across the bridge was also investigated. Interestingly, all treatment groups at six weeks resulted in numerous betaIII tubulin-positive axons penetrating the bridge from the rostral stump but stopping as they approached the caudal stump, as illustrated in Figure 5A. These axons were not GFP-positive, indicating that they originated from the host. Double labelling of betaIII-tubulin with synaptophysin, a neural synaptic marker, shows that there is some association between the endogenous axons and the transplanted GFP-positive cells (Figure 5B). Also present in the bridge are RECA1 positive endothelial cells, which form discrete blood vessels as early as two-weeks (Figure 5C). No apparent association was found between blood vessel formation and treatment, or blood vessel vicinity to surviving NSPCs. Apart from the GFP-positive transplanted NSPCs, the tissue bridge was composed mainly of prolyl-4-hydroxylase-positive, collagen-producing fibroblasts at 6 weeks (Figure 5D). GFP-positive transplanted NSPCs were negative for the fibroblast marker.

## Discussion

In this study, we aimed to understand the role of prolonged dbcAMP exposure on neuronal differentiation and the impact of pre-differentiated vs. *in situ* differentiated neurons on their survival and integration *in vivo*. By using the fully transected spinal cord injury model, we could clearly follow the effect of differentiated stem cells on tissue regeneration and locomotor functional recovery. The entubulation strategy, with which we have significant experience [9], [10], [33], [34], is most suitable for these full-transection studies. The incorporation of dbcAMP-releasing microspheres in the tube walls allowed us to investigate the timing of differentiation on survival.

Interestingly, enhanced neuronal differentiation was observed with only prolonged, 7 d dbcAMP exposure and thus necessitated the inclusion of a prolonged release strategy in the chitosan tubular construct design. PLGA microspheres were fabricated and tailored for short-term release of dbcAMP and embedded into chitosan channels. In order to facilitate 7 d *in vitro* cultures, NSPCs were encapsulated in fibrin scaffolds that were incorporated into the channels. While previous studies have had NSPCs seeded along the interior walls of the channels [9], [10], this was impractical for prolonged *in vitro* culture where cell proliferation resulted in cells dislodging from the channel surface. Fibrin scaffolds not only provided better entrapment of NSPCs, but also resulted in improved consistency in cell number and distribution. Fibrin scaffolds form quickly from fibrinogen and thrombin, and have been shown to be safe for cells in culture [35] and *in vivo* [36]. After spinal cord injury, fibrin scaffolds have been shown to attenuate glial scar formation [37]. Moreover, we have previously shown that guidance channels filled with fibrin matrix promote axonal regeneration and functional recovery after spinal cord transection [34]. The cell differentiation data of NSPCs cultured in fibrin-filled, microsphere-loaded channels for 7 days *in vitro* confirmed that NSPCs are still dbcAMP-responsive in 3D fibrin scaffolds. More importantly, the NSPCs preferentially differentiated into neurons when cultured in channels where microspheres provided the only source of dbcAMP, validating microsphere-loaded channels as an effective drug delivery system *in vitro*. Although the neuronal differentiation percentage with dbcAMP on fibrin scaffolds was not as high as that on 2D chitosan films (∼50% vs. ∼80%), the effect was still a marked increase over non-dbcAMP treated cells (7%). It is possible that a higher target dose of dbcAMP may compensate for this decrease in neuronal differentiation, but this was not investigated. Cell fate determination is a complex process that not only depends on presence of soluble factors and time, but is also influenced by surface chemistry/adhesion [38] and mechanical properties [31], [39], so it was important to confirm that NSPCs would respond to dbcAMP in the three-dimensional fibrin matrix.

To better understand the benefit of pre-differentiated neurons vs. *in situ* (via dbcAMP-releasing microspheres), neuronal differentiation and survival were studied *in vivo*, in a fully-transected SCI rat model. Untreated NSPCs served as controls. Unexpectedly, NSPCs pre-differentiated with dbcAMP prior to transplantation resulted in a striking increase in cell survival of 80% whereas both untreated and dbcAMP-microsphere-treated NSPCs had survival rates of approximately 15%. There were no significant changes in survival of transplanted NSPCs between two and six weeks within a given treatment. Paired with low level of staining with Ki67 proliferative marker, this suggests that the NSPC population had stabilized between these two time points. The enhanced survival effect associated with the dbcAMP 4 div group is attributed directly to pre-treatment with dbcAMP, as this was not observed with *in situ* dbcAMP delivery (dbcAMP-MS 1 div) nor in matching untreated controls (untreated 4 div) which would account for the extended *in vitro* incubation time including scaffold integrity, cell distribution, adhesion, or other factors.

Survival effects have been previously associated with dbcAMP pre-treatment, which can provide neuroprotection against excitotoxicity [40]. Rolipram, a phophodiesterase-IV inhibitor that works primarily by increasing intracellular cAMP [41], has been shown to be neuroprotective in the acute phase of SCI [42], [43]. Moreover, downstream pathways of cyclic-AMP are important for the survival of newly generated neurons during development [44]. Recently, Nout et al. used combined rolipram and dbcAMP drug treatment with glial restricted precursor cell transplants and observed reduced transplant survival with dbcAMP treatment [45]. However, the concentration of dbcAMP used in that study was 50 mM. Concentrations of 1 mM, as used in this study, are well-tolerated in the spinal cord whereas high doses can be damaging [46]. Rolipram-based elevation of cAMP has been shown to increase survival of transplanted olfactory ensheathing cells [43], and in combination with Schwann cell grafts, was implicated in axonal sparing [47]. The present study shows no effect on transplant survival with *in situ* dbcAMP delivery compared to no dbcAMP treatment, suggesting that enhanced transplant survival resulted from either a priming or pre-differentiation effect.

Pre-differentiation or pre-commitment of stem cells down a specific lineage may account for greater survival. Tarasenko et al. reported marginally higher survival when neuronally committed progenitor cells versus undifferentiated cells were transplanted in the injured CNS [48]. Olstorn et al. transplanted two preparations of human NSPCs in ischemic brain, demonstrating that both undifferentiated and neuronally-committed NSPCs were able to migrate towards the lesion site [49]. However, no differences in survival between the two preparations were reported. Davies et al. pre-treated glial restricted precursors with bone morphogenic protein-4 (BMP4) to derive immature astrocytes and explored the differences between the two populations after transplantation into lesioned rat spinal cord [50]. They found that pre-differentiated astrocytes better integrated with host regenerating tissue by supporting axonal regeneration and reducing astrogliosis. Interestingly, this group found that pre-differentiated astrocytes from ciliary neurotrophic factor (CNTF)-treated precursors had a detrimental effect [51] versus the beneficial effect observed with BMP4, indicating that subtle differences in the pre-differentiated progeny can significantly alter *in vivo* behaviour. Committed oligodendrocytes also performed better than undifferentiated NSPCs, showing better ability to remyelinate denuded spinal cord axons [52]. However, pre-differentiation of NSPCs to neurons is not always beneficial. For example, neuronal pre-differentiation resulted in poorer engraftment and survival than that achieved with undifferentiated NSPCs in hippocamppal transplant studies for Parkinson's disease [53], [54]. The extent of differentiation or perhaps maturation down a specific lineage may be of great importance. The 4 div with dbcAMP exposure ensured differentiation down the neuronal lineage; however, the differentiated progeny are unlikely fully mature, thereby promoting their survival and integration with the host tissue. This committed, but not mature, phenotype for enhanced survival *in vivo* may allow greater integration.

Pre-treatment of dbcAMP resulted in the highest number of betaIII-tubulin positive neurons after two weeks *in vivo*, while the lowest percentage of neurons was associated with cells not exposed to dbcAMP, whether cultured for 1 or 4 div prior to transplant. The increased percentage of neurons in dbcAMP pre-treated NSPCs came at the expense of oligodendrocyte and astrocyte differentiation, which were both higher in untreated NSPCs. Although not statistically significant, *in situ* delivery of dbcAMP via microspheres consistently resulted in differentiation values that were between dbcAMP pre-treated and untreated groups. This suggests that dbcAMP-microspheres were likely able to influence NSPC fate *in vivo*, but that certain parameters of the delivery might be lacking, for example drug concentration or length of administration. Indeed, release parameters of PLGA microspheres can differ between an *in vitro* and *in vivo* environment. Using radio-labelling, de Boer et al. showed linear but more rapid release when comparing *in vitro* and *in vivo* release profiles of nerve growth factor from PLGA microspheres [55]. Technical restraints limited that study to a subcutaneous model, and indeed even more rapid degradation and release would be expected in injured tissue where acidic pH would cause accelerated degradation of the PLGA.

The tissue bridge had a high density of cells, the majority of which were collagen-producing fibroblasts, likely of meningeal origin from the disrupted pia, arachnoid, and dura. Angiogenesis was apparent as early as two weeks throughout the bridge, as evidenced by RECA1-postive staining of blood vessels. Notably, endogenous axonal regeneration into the bridge was observed in all treatment groups, and all showed initiation from the rostral stump of the spinal cord. However, the axons encountered a barrier at the caudal end of the bridge, from the glial scar which prevented penetration into the caudal stump of the spinal cord.

Complete spinal cord transection is the most severe injury model that results in zero hindlimb movement immediately after injury. Typically rats will spontaneously recover only a very limited range of motion in the lower limbs over time. In this study, we showed that animals receiving dbcAMP pre-treated NSPCs showed a small but statistically significant (p<0.05) improvement in hindlimb function resulting in some movement of hindlimb joints. A strong histological basis for this improvement is not apparent, as endogenous axonal regeneration across the tissue bridge did not appear to be qualitatively different among treatment groups. It is possible that some of the surviving NSPCs which spanned the entire length of the bridges, contributed to the observed enhanced functional recovery. Indeed, these transplanted cells appear to associated closely with each other to form a continuous network (Figure 3C,D). Directed differentiation of NSPCs into neurons has been reported by others to improve functional outcome after experimental SCI [56], [57]. We did observe synapse formation between host axons and surviving NSPCs, which may underlie this minimal recovery if the axons from these surviving transplanted cells reached any of the motor neurons in the distal spinal cord. However, the apparent loss of neuronal phenotype of our transplanted NSPCs at six weeks suggests that a neuronal relay in the traditional sense may not be the mechanism responsible for the observed enhanced function. Additional locomotor recovery would have likely continued in all groups at longer timepoints as BBB scores had not yet plateaued after six weeks. Thus, it is possible that dbcAMP pre-treatment does not enhance long-term functional outcome, but only accelerates the rate of recovery.

Cell transplant strategies offer great potential for replacing lost and damaged tissue. Drug treatments and biomaterial scaffolds further improve the efficacy of transplant cell survival and differentiation, as well as promote endogenous tissue regeneration. Our entubulation strategy combines these three aspects of treatment and has shown to be efficacious in promoting NSPC survival and host axonal regeneration.
